# Satisfaction of family members with inpatient psychiatric care and its correlates: a national survey in China

**DOI:** 10.1186/s12888-019-2362-6

**Published:** 2019-12-30

**Authors:** Feng Jiang, Linlin Hu, Ruiping Zhao, Huixuan Zhou, Yinuo Wu, Jeffrey J. Rakofsky, Tingfang Liu, Huanzhong Liu, Yuanli Liu, Yi-Lang Tang

**Affiliations:** 1 School of public health, Chinese Academy of Medical Sciences and Peking Union Medical Colleg, No.3 Dong Dan San Tiao, Dongcheng District, Beijing, China; 2 Department of doctor-patient relationship, Peking University People’s Hospital, No.11 Xizhimeng South Road, Xicheng District, Beijing, China; 3 Department of Psychiatry and Behavioral Sciences, Emory University, 12 Executive Park Drive NE, Suite 348, Atlanta, GA USA; 4 Institute for Hospital Management of Tsinghua University, No.30 Shuangqing Road, Haidian District, Beijing, China; 5 Department of Psychiatry, Chaohu Hospital of Anhui Medical University, No.64 Chaohu North Road, Chaohu District, Hefei, China; 6 Atlanta VA Medical Center, 1670 Clairmont Road, Decatur, GA USA

**Keywords:** Family satisfaction, Individual characteristics, Related factors, Psychiatric hospitals, China

## Abstract

**Background:**

Measuring family members’ satisfaction with inpatient psychiatric care may help improve the quality of healthcare in psychiatric hospitals. This survey aimed to investigate the satisfaction of family members with inpatient psychiatric care and to explore its associated factors, using a newly-developed 5-item questionnaire.

**Methods:**

This study included 1598 family members of psychiatric inpatients in 32 tertiary public psychiatric hospitals in 29 provinces of China. Satisfaction and demographic data were collected by research staff while patient and hospital data were retrieved separately.

**Results:**

We found that the overall satisfaction level was 93.84% (23.46/25). The total satisfaction score in Northeast China was the highest, followed by the East, Middle and West regions (*p* < 0.001). There was no significant sex difference in total family satisfaction scores. Family members with a lower educational background (elementary school or less) had significantly lower satisfaction. Family members of patients who were diagnosed with schizophrenia were significantly less satisfied with doctor-family communication. In different treatment response subgroups, the marked improvement subgroup had significantly higher total satisfaction scores and subscores. Meanwhile, lower self-payment expenses and a higher number of psychologic treatments offered per day were significantly associated with higher total satisfaction scores and all subscores. Logistic regression showed a higher educational background, more psychologic treatments offered per day, adequacy of professional staffing (higher doctor/bed, nurse/bed and psychologist/bed ratio) were all significantly associated with higher family satisfaction.

**Conclusions:**

We suggest government and hospital managers recruit more mental health professions to improve family satisfaction. If feasible, providing more psychologic treatments to inpatients may also improve families’ satisfaction and involvement.

## Background

The satisfaction of patients and their families is an important quality indicator of hospital healthcare [1]. Since patients’ families are key stakeholders and important participants of the healthcare process, monitoring and improving family satisfaction is both relevant and necessary. Measuring the satisfaction of family members and how they perceive the care provided to patients may help improve hospital healthcare quality [2, 3]. For mental health care, the involvement of family members in patients’ care is very important for treatment and recovery, sometimes of critical importance [4–7]. This is even more so as most Chinese families are expected to be involved in patients’ healthcare, including important decision making [8, 9]. A 2001 WHO Report recommends that psychiatric patients’ family members should be involved in mental health planning and practice, especially in the treatment process [10]. One study showed satisfied family members improved the treatment for psychiatric patients and are of the utmost importance for the mental healthcare evaluation [11].

Although quite a few studies have been published regarding parents’ satisfaction with inpatient psychiatric care [12–16], few studies have focused on the satisfaction of family members with the inpatient psychiatric care of adults. In 2002, Gigantesco et al. interviewed 265 relatives of psychiatric patients in Italy to measure their satisfaction, and they used a self-developed 11-item questionnaire, including information on services, environment privacy, involvement in the treatment program, etc. They invited all family members who had visited inpatients to participate and 105 relatives of psychiatric inpatients were recruited. The overall satisfaction level was 69.2%. The most reported reason for dissatisfaction was the lack of information about treatment. They found being female was associated with less satisfaction among relatives. Other factors, such as relatives’ age, relatives’ education level (> 8 years or not), or a patient diagnosis of psychosis, were not associated with dissatisfaction among relatives [17]. In 2013, Macinnes et al. interviewed 63 carers of forensic inpatients via telephone in United Kingdom, and they also used a self-developed 8-item questionnaire, including information provided by the service, involvement in care, ward environment, discharge plans, among others. They found that the overall satisfaction was 78%. Similar to the findings of Gigantesco et al. (2002), delivering appropriate information was strongly associated with carers’ satisfaction. Additionally, they found that parents were more dissatisfied than other carers [18].

Dourado et al. (2018) evaluated the satisfaction of 80 Brazilian family members for psychiatric inpatient service. They used the Brazilian Mental Health Services’ Family Satisfaction Scale, which was made up of eight quantitative questions. The instrument was designed on a 5-point Likert scale, and it included questions about satisfaction with various aspects of health care, such as treatment results, reception, staff competence, and privacy protection. They found the mean overall satisfaction score was 4.05/5, or 81%, indicating a high satisfaction level. They found no significant association between family satisfaction and participants’ sociodemographic variables, such as age, educational level, and kinship degree [19].

So far, only two such studies have been published in China, investigating the family satisfaction of psychiatric patients. Shi et al. (2004) surveyed 249 family members of psychiatric inpatients in Shanghai. They used the revised mental health service scale, which contained 58 items, including involvement in the treatment, information about services, friendliness of mental health professions, listening skills, privacy protection, etc. They found 77.1% of participants were satisfied and the most important reason for dissatisfaction was their lack of involvement in patients’ treatment decisions [20]. However, this study did not include many important variables related to hospitalization (such as length of stay, costs, treatment, etc.) or hospital-level variables. In 2010, Zhang et al. surveyed 196 family members in Hangzhou city (the capital city of Zhejiang province in east China) about their satisfaction with nursing services in a psychiatric hospital. Using a 24-item questionnaire, including questions about nursing skills, psychological nursing, environment, service etiquette, etc., they found the overall satisfaction level was 84% with significant differences in sex, educational background, and home income. Male sex, lower educational background, and lower home income were all associated with higher satisfaction [21]. Similar to the first one [20], the two surveys only focused on the family members without considering factors related to the patient involved or the inpatient treatment. Furthermore, those instruments were designed for families only and were not appropriate for patients.

Based on the above, it is important to develop a brief and reliable instrument that can be used by both patients and family members, to measure the level of satisfaction with inpatient psychiatric services. This survey was designed with the following goals: (1) To develop a brief and reliable instrument to measure patients’ and families’ satisfaction with psychiatric inpatient care; (2) To examine the differences among various hospitals and regions; (3) To examine the correlates with family satisfaction, including patient factors, treatment-related factors, and hospital-level factors. We hope the data may offer some insight into factors contributing to family members’ dissatisfaction and ways to modify them, ultimately improving the quality of patient-healthcare.

## Methods

### Samples

This study was one part of a larger project, the National Survey for the Evaluation of Psychiatric Hospital Performance [22, 23]. In this project, we included 32 tertiary public psychiatric hospitals, which were in the capital cities of 29 provinces. Family members of psychiatric inpatients, who were discharged from December 25 to 27 in 2017, were included. However, we invited only one family member for each inpatient on the day of discharge. The participants should meet the flowing criteria: was involved in the patient’s care, no less than 18 years old, understood the survey questions and signed the informed consent. The family members were evaluated by local research staff who was not involved in the patient’s treatment. At the same time, the discharged inpatients were also surveyed to determine their satisfaction with the inpatient service [23].

### Measures

At the time of the study, there were no appropriate instruments available in the Chinese language to measure the satisfaction of family members of psychiatric patients. Therefore, we developed a local questionnaire for both patients and families, based on a literature search and expert discussions [24–26]. Findings from previous studies suggested that the common reasons for dissatisfaction of family members include aspects related to the doctor-patient relationship, technical skills of healthcare, the health-care institution, billing and insurance, and lack of information or communication [27].

Family members’ demographic information was based on self-report, including sex, age, relationship with patients, educational background. Patients’ clinical characteristics were collected by research staff, including psychiatric diagnosis according to *the International Classification of Diseases and Related Health Problems 10th revision* (ICD-10) [28], Global Assessment of Functioning (GAF) scale score at admission [29], treatment response based on the Clinical Global Impression (CGI) scale [30], length of stay (LOS, days), number of psychological treatments, and number of patients who received electroconvulsive therapy (ECT) during the hospitalization. The payment type, total cost, self-pay cost, whether it was their first psychiatric hospitalization, and whether the hospitalization was involuntary were also recorded.

We retrieved each hospital’s data from the Hospital Information System (HIS), including the number of beds, doctors (primarily psychiatrists as these are free-standing psychiatric hospitals), nurses, and psychologists.

### Statistical analysis

Descriptive analyses were used to describe the data. The total satisfaction score and subscores were treated as continuous variables. Other variables were treated as categorical variables. Comparisons of total satisfaction scores and subscores were calculated using the Mann-Whitney U test or Kruskal-Wallis test, as appropriate. Associations between family satisfaction scores and the related factors were analyzed using the Spearman’s correlation tests.

Then, the total satisfaction score was transformed into a binary categorical variable based on the mean score. Cases with a higher score than the mean score were categorized into the satisfied group, and those below the mean were categorized into the dissatisfied group. In this study, as respondents were nested in 32 hospitals, multi-level multiple logistic regression was used to examine the relationship between total satisfaction scores and factors involved in the analysis, which allowed for association across respondents within hospitals [31]. Stata 15 (StataCorpLP, College Station, TX, USA) was used for these statistical analyses. All of the tests were two-sided and the statistical significance was defined as *p* < 0.05.

## Results

### Development of the questionnaire to measure satisfaction with inpatient psychiatric services

The final version of the questionnaire consisted of five closed questions about communication, privacy protection, medical services, cost, and general satisfaction (the questionnaire is available upon request). Each item was measured on a 5-point Likert scale (1 = very dissatisfied, 5 = very satisfied). The questionnaire provided four subscores to cover different aspects of psychiatric care, plus one subscore for general satisfaction. In the meantime, adding all five subscores would yield a total score. The questionnaire was drafted by Feng Jiang and Yi-Lang Tang and then revised by other coauthors (Huanzhong Liu, Yuanli Liu). Later, it was distributed among a small group of mental health professionals to obtain feedback. A revised version was completed based on the feedback. Then, it was pilot tested in a small sample (*N* = 51), and some of the words were revised again based on the feedback. In the study sample, the Cronbach’s α coefficient for the family satisfaction questionnaire was 0.91, and the reliability of test-retest in a pilot study was 0.75.

### Description of sample characteristics and related factors

In total 1780 psychiatric inpatients and 1780 family members participated in the survey across China, 182 cases were excluded due to missing data. Finally, data from 1598 family members were included in the analysis. On average, each hospital had 50 participants and ranged from 9 to 94.

The participants’ demographic features are shown in Table 1. The median of age was 47.7 years old, and nearly one third (31.2%) were between 41 and 50 years old. 53.7% were male, 35.0% were patient’s parents, and the majority (58.3%) had received a high school education or beyond.

**Table 1:** Individual characteristics of family members, *n* (%)

Variables	Family members (*n* = 1598)
Sex	
Male	858 (53.69)
Female	740 (46.31)
Age^a^	
≤ 30	166 (10.39)
31–40	299 (18.71)
41–50	498 (31.16)
51–60	403 (25.22)
>60	232 (14.52)
Relationship with patients	
Parents	560 (35.04)
Spouses	460 (28.79)
Offspring	213 (13.33)
Sibling	240 (15.02)
Others	125 (7.82)
Educational background	
Elementary school	257 (16.08)
Middle school	410 (25.66)
High school	528 (33.04)
College education	374 (23.40)
Graduate education	29 (1.81)

^a^The median age of the family members was 48 years old, the inter-quartile range (IQR) was 17

The patient’s characteristics are shown in Table 2. Their mean age was 41.9 ± 15.5 years old. 48.0% were male, 44.1% were hospitalized for the first time, 43.9% were admitted involuntarily, 37.1% had a LOS of 21–40 days, 47.4% had a diagnosis of schizophrenia or related disorders, 41.2% had a GAF score of 41–60 at admission, 8.0% were placed in seclusion at least once, 27.35% were put in restraints at least once, 14.6% had received ECT treatment, 36.5% had received psychological treatment 0.5–1.0 times per day, and 57.9% were rated “marked improvement” on the day of discharge. Total expense related to hospitalization ranged from 400 to 31,9878 RMBs (median = 15,952), and the total amount of self-payment ranged from 0 to 26,7631 RMBs (median = 3654).

**Table 2:** Related characteristics of patients, *n* (%)

Variables	Patients
Sex	
Male	767 (48.00)
Female	831 (52.00)
Insurance-pay	
Yes	1215 (76.03)
No	383 (23.97)
Frist time of psychiatric hospitalization	
Yes	705 (44.12)
No	893 (55.88)
Involuntary admission	
Yes	701 (43.86)
No	897 (56.13)
Length of stay (days)^a^	
≤ 20	506 (25.41)
21–40	593 (37.11)
41–60	274 (17.15)
>60	325 (20.34)
Diagnosis	
Schizophrenia and related disorders	757 (47.37)
Mood disorder	483 (30.23)
Others diagnosis	358 (22.40)
GAF of admission^b^	
≤ 20	283 (17.71)
21–40	352 (22.03)
41–60	659 (41.24)
>60	304 (19.02)
ECT treatment^c^	
0	1364 (85.36)
≥ 1	234 (14.64)
Treatment response	
Marked improvement	925 (57.88)
Improvement	591 (36.98)
Somewhat improvement	73 (4.57)
No change or worse	9 (0.56)
Number of Psychologic treatment per day^d^	
≤ 0.4	894 (55.94)
0.5–1.0	583 (36.48)
>1.0	121 (7.57)
Total payment (RMBs)^e^	
≤ 10,000	471 (29.47)
10,001–20,000	540 (33.79)
20,001–30,000	286 (17.90)
>30,000	301 (18.84)
Self-payment (RMBs)^f^	
≤ 5000	919 (57.51)
5001–10,000	305 (19.09)
10,001–20,000	209 (13.08)
>20,000	165 (10.33)

^a^The median LOS is 33 days, the inter-quartile range (IQR) is 34

^b^The median GAF is 45, the inter-quartile range (IQR) is 31

^c^The median ECT is 0, the inter-quartile range (IQR) is 0

^d^The median is 0.30, the inter-quartile range (IQR) is 0.59

^e^The median is 15,952, the inter-quartile range (IQR) is 17,008

^f^The median is 3654, the inter-quartile range (IQR) is 8233

One US dollar ≈ 6.8 RMBs at the time of the study

At the hospital level, the doctor/bed ratios ranged from 0.05 to 0.32 (median = 0.17). The nurse/bed ratios ranged from 0.14 to 0.64 (median = 0.38), and psychologist/bed ratios ranged from 0 to 0.42 (median = 0.01).

### Family satisfaction scores and related factors

The mean and standard deviation total family satisfaction score was 23.46 ± 2.47 out of 25, and the median total score of the respondents was 25. The inter-quartile range (IQR) was 3. Families were least satisfied with the cost of hospitalization, with a score of 4.58 ± 0.66 out of 5. The median (IQR) of the satisfaction score for hospitalization cost was 5(1). On other items, the subscores were comparable to each other: 4.72 ± 0.52 for general satisfaction, 4.72 ± 0.52 for medical services satisfaction, 4.72 ± 0.53 for privacy protection satisfaction, and 4.71 ± 0.55 for doctor-family communication satisfaction. The median (IQR) was 5(1), 5(1), 5(1), 5(1), respectively.

There were significant differences in total satisfaction scores and in the subscores among the 32 hospitals (*p* < 0.001). At the regional level, the total satisfaction score in Northeast China (24.51) was the highest, followed (in descending order) by the East (23.79), Middle (23.30) and West (22.53) regions (*p* < 0.001). This trend in regional differences is also seen among the subscores. For the general satisfaction dimension, the means of Northeast, East, Middle, and West China regions were 4.89, 4.79, 4.69, and 4.53 (*p* < 0.001), respectively. For the hospitalization cost score, the means of Northeast, East, Middle, and West China were 4.88, 4.65, 4.55, and 4.36 (*p* < 0.001). For the medical services score, the means of Northeast, East, Middle, and West China were 4.93, 4.78, 4.70, and 4.54 (*p* < 0.001), respectively. For the privacy protection score, the means of Northeast, East, Middle, and West were 4.91, 4.79, 4.68, and 4.56 (*p* < 0.001), respectively. For doctor-family communication, the means of Northeast, East, Middle, and West were 4.90, 4.78, 4.69, and 4.54 (*p* < 0.001), respectively.

Compared with the satisfaction level of the inpatients [23], family members had a significantly higher total satisfaction score, and higher scores for the hospitalization cost dimension and doctor-patient/family communication dimension. The mean score of the family total satisfaction score was 23.46 in families vs. 23.35 in inpatients (*p* < 0.05). For the hospitalization cost dimension, the score was 4.58 in families vs. 4.55 in patients, *p* < 0.05, and for the doctor-patient/family communication dimension, the score was 4.71 in families vs. 4.68 in patients, *p* < 0.05 (see Table 3).

**Table 3:** Analysis of satisfaction score and related factors (mean ± standard deviation)

	Total satisfaction score		General satisfaction score		Costs satisfaction score		Medical services satisfaction score		Privacy protection satisfaction score		Doctor-family communication satisfaction score	
Sex^a^		*P* = 0.27		*P* = 0.48		*P* = 0.49		*P* = 0.95		*P* = 0.07		*P* = 0.11
Male	23.52 ± 2.48		4.72 ± 0.51		4.59 ± 0.67		4.72 ± 0.54		4.74 ± 0.51		4.73 ± 0.55	
Female	23.38 ± 2.44		4.70 ± 0.51		4.57 ± 0.66		4.71 ± 0.50		4.69 ± 0.54		4.69 ± 0.56	
Age^b^		*P* = 0.65		*P =* 0.95		*P* = 0.32		*P* = 0.69		*P* = 0.83		*P* = 0.45
≤ 30	23.19 ± 2.62		4.71 ± 0.50		4.48 ± 0.78		4.67 ± 0.54		4.68 ± 0.56		4.64 ± 0.66	
31–40	23.55 ± 2.37		4.72 ± 0.51		4.61 ± 0.63		4.74 ± 0.48		4.73 ± 0.49		4.73 ± 0.52	
41–50	23.46 ± 2.49		4.70 ± 0.52		4.58 ± 0.64		4.71 ± 0.55		4.73 ± 0.54		4.73 ± 0.57	
51–60	23.36 ± 2.41		4.71 ± 0.51		4.58 ± 0.67		4.72 ± 0.50		4.71 ± 0.52		4.70 ± 0.52	
>60	23.50 ± 2.51		4.73 ± 0.52		4.67 ± 0.64		4.70 ± 0.54		4.73 ± 0.51		4.71 ± 0.53	
Educational level^b^		*P* = 0.03		*P* = 0.02		*P* = 0.01		*P* < 0.01		*P* = 0.03		*P* = 0.02
Elementary school	22.98 ± 2.50		4.64 ± 0.54		4.47 ± 0.64		4.59 ± 0.58		4.63 ± 0.54		4.63 ± 0.55	
Middle school	23.37 ± 2.50		4.69 ± 0.51		4.55 ± 0.71		4.71 ± 0.51		4.70 ± 0.54		4.70 ± 0.56	
High school	23.56 ± 2.54		4.72 ± 0.53		4.63 ± 0.61		4.75 ± 0.46		4.75 ± 0.51		4.74 ± 0.51	
College	23.61 ± 2.49		4.74 ± 0.50		4.63 ± 0.64		4.76 ± 0.51		4.74 ± 0.55		4.72 ± 0.61	
Graduate education	24.44 ± 1.35		4.89 ± 0.30		4.82 ± 0.38		4.89 ± 0.30		4.86 ± 0.35		4.96 ± 0.18	
Relationship with patients^b^		*P* = 0.32		*P* = 0.23		*P* = 0.21		*P* = 0.74		*P* = 0.37		*P* = 0.38
Parents	23.42 ± 2.35		4.70 ± 0.50		4.55 ± 0.66		4.71 ± 0.50		4.73 ± 0.49		4.72 ± 0.48	
Spouses	23.44 ± 2.58		4.71 ± 0.53		4.59 ± 0.69		4.72 ± 0.52		4.72 ± 0.54		4.69 ± 0.60	
Offspring	23.32 ± 2.89		4.68 ± 0.59		4.57 ± 0.72		4.69 ± 0.59		4.68 ± 0.58		4.68 ± 0.65	
Sibling	23.45 ± 2.31		4.71 ± 0.49		4.58 ± 0.63		4.72 ± 0.54		4.71 ± 0.55		4.72 ± 0.59	
Others	23.89 ± 1.99		4.81 ± 0.38		4.71 ± 0.50		4.77 ± 0.43		4.80 ± 0.43		4.79 ± 0.44	
Insurance-pay^a^		*P* = 0.58		*P* = 0.39		*P* = 0.14		*P* = 0.77		*P* = 0.85		*P* = 0.84
Yes	23.47 ± 2.44		4.72 ± 0.51		4.59 ± 0.64		4.72 ± 0.51		4.72 ± 0.53		4.71 ± 0.56	
No	23.39 ± 2.55		4.69 ± 0.53		4.54 ± 0.73		4.71 ± 0.54		4.72 ± 0.51		4.72 ± 0.52	
Frist time of psychiatric hospitalization^a^		*P* = 0.21		*P* = 0.11		*P* = 0.11		*P* = 0.82		*P* = 0.47		*P* = 0.18
Yes	23.37 ± 2.63		4.69 ± 0.55		4.55 ± 0.71		4.71 ± 0.53		4.71 ± 0.53		4.69 ± 0.58	
No	23.52 ± 2.33		4.73 ± 0.48		4.60 ± 0.62		4.72 ± 0.51		4.73 ± 0.52		4.73 ± 0.53	
Involuntary admission^a^		*P* = 0.27		*P* = 0.56		*P* = 0.83		*P* = 0.23		*P* = 0.10		*P* = 0.16
Yes	23.52 ± 2.50		4.72 ± 0.52		4.58 ± 0.68		4.73 ± 0.53		4.74 ± 0.50		4.73 ± 0.55	
No	23.39 ± 2.44		4.70 ± 0.50		4.58 ± 0.64		4.70 ± 0.53		4.70 ± 0.54		4.69 ± 0.55	
Diagnosis^b^		*P* = 0.30		*P* = 0.66		*P* = 0.55		*P* = 0.76		*P* = 0.20		*P* = 0.04
Schizophrenia and related disorders	23.47 ± 2.39		4.72 ± 0.50		4.58 ± 0.65		4.71 ± 0.51		4.72 ± 0.49		4.72 ± 0.53	
Mood disorder	23.55 ± 2.25		4.71 ± 0.48		4.60 ± 0.65		4.73 ± 0.48		4.74 ± 0.46		4.75 ± 0.47	
Others diagnosis	23.29 ± 2.88		4.69 ± 0.57		4.55 ± 0.71		4.70 ± 0.60		4.68 ± 0.65		4.65 ± 0.70	
Treatment response^b^		*P* < 0.01		*P* = 0.01		*P* = 0.06		*P* < 0.01		*P* = 0.03		*P* < 0.01
Marked improvement	23.62 ± 2.36		4.74 ± 0.48		4.61 ± 0.65		4.76 ± 0.47		4.75 ± 0.50		4.75 ± 0.53	
Improvement	23.16 ± 2.63		4.66 ± 0.55		4.52 ± 0.68		4.64 ± 0.59		4.67 ± 0.56		4.64 ± 0.60	
Somewhat improvement	23.07 ± 2.28		4.61 ± 0.48		4.43 ± 0.63		4.62 ± 0.53		4.65 ± 0.46		4.55 ± 0.43	
No change or worse	22.45 ± 1.01		4.46 ± 0.43		4.37 ± 0.49		4.57 ± 0.52		4.58 ± 0.33		4.52 ± 0.55	
Length of stay (days)^b^		*P* = 0.59		*P* = 0.44		*P* = 0.47		*P* = 0.53		*P* = 0.67		*P* = 0.63
≤ 20	23.53 ± 2.46		4.74 ± 0.48		4.62 ± 0.64		4.73 ± 0.52		4.72 ± 0.57		4.71 ± 0.58	
21–40	23.50 ± 2.52		4.71 ± 0.53		4.58 ± 0.67		4.73 ± 0.52		4.74 ± 0.51		4.73 ± 0.55	
41–60	23.41 ± 2.21		4.69 ± 0.49		4.56 ± 0.63		4.69 ± 0.51		4.72 ± 0.47		4.72 ± 0.51	
>60	23.31 ± 2.58		4.69 ± 0.54		4.54 ± 0.70		4.69 ± 0.52		4.69 ± 0.53		4.68 ± 0.57	
GAF at admission^b^		*P* = 0.85		*P* = 0.70		*P* = 0.88		*P* = 0.72		*P* = 0.89		*P* = 0.66
≤ 20	23.53 ± 2.33		4.74 ± 0.46		4.56 ± 0.68		4.75 ± 0.46		4.74 ± 0.50		4.72 ± 0.55	
21–40	23.55 ± 2.41		4.73 ± 0.51		4.61 ± 0.64		4.72 ± 0.52		4.73 ± 0.48		4.74 ± 0.51	
41–60	23.39 ± 2.63		4.69 ± 0.55		4.57 ± 0.69		4.70 ± 0.54		4.71 ± 0.56		4.69 ± 0.59	
>60	23.42 ± 2.30		4.71 ± 0.48		4.56 ± 0.62		4.70 ± 0.55		4.71 ± 0.51		4.71 ± 0.53	
ECT treatment^a^		*P* = 0.38		*P* = 0.71		*P* = 0.14		*P* = 0.25		*P* = 0.64		*P* = 0.74
0	23.48 ± 2.46		4.71 ± 0.51		4.59 ± 0.66		4.72 ± 0.52		4.72 ± 0.52		4.71 ± 0.56	
≥1	23.32 ± 2.51		4.70 ± 0.52		4.52 ± 0.69		4.68 ± 0.54		4.70 ± 0.51		4.70 ± 0.52	
Total payment (RMBs)^b^		*P* = 0.10		*P* = 0.07		*P* = 0.04		*P* = 0.53		*P* < 0.01		*P* = 0.24
≤ 10,000	23.64 ± 2.25		4.74 ± 0.48		4.64 ± 0.59		4.74 ± 0.49		4.75 ± 0.49		4.74 ± 0.52	
10,001–20,000	23.26 ± 2.59		4.67 ± 0.54		4.55 ± 0.69		4.69 ± 0.53		4.66 ± 0.57		4.67 ± 0.57	
20,001–30,000	23.47 ± 2.38		4.72 ± 0.51		4.51 ± 0.70		4.71 ± 0.51		4.77 ± 0.46		4.73 ± 0.54	
>30,000	23.49 ± 2.64		4.73 ± 0.53		4.59 ± 0.68		4.72 ± 0.56		4.72 ± 0.54		4.72 ± 0.59	
Self-payment (RMBs)^b^		*P* < 0.01		*P* < 0.01		*P* < 0.01		*P* < 0.01		*P* = 0.01		*P* < 0.01
≤ 5000	23.79 ± 3.30		4.78 ± 0.46		4.64 ± 0.58		4.78 ± 0.46		4.77 ± 0.48		4.76 ± 0.52	
5001–10,000	23.73 ± 2.24		4.73 ± 0.48		4.62 ± 0.64		4.73 ± 0.49		4.73 ± 0.51		4.74 ± 0.52	
10,001–20,000	23.59 ± 2.27		4.63 ± 0.52		4.40 ± 0.87		4.63 ± 0.55		4.69 ± 0.46		4.67 ± 0.48	
>20,000	23.03 ± 2.47		4.58 ± 0.66		4.40 ± 0.77		4.61 ± 0.69		4.62 ± 0.66		4.56 ± 0.75	
Number of Psychologic treatment per day^b^		*P* < 0.01		*P* < 0.01		*P* < 0.01		*P* = 0.09		*P* = 0.02		*P* < 0.01
≤ 0.4	23.28 ± 2.69		4.68 ± 0.56		4.53 ± 0.71		4.69 ± 0.56		4.69 ± 0.56		4.67 ± 0.59	
0.5–1.0	23.65 ± 2.14		4.74 ± 0.46		4.62 ± 0.68		4.73 ± 0.48		4.74 ± 0.48		4.76 ± 0.48	
>1.0	23.83 ± 2.12		4.81 ± 0.40		4.65 ± 0.56		4.80 ± 0.42		4.82 ± 0.40		4.78 ± 0.59	

^a^t test; ^b^ANOVA

### Multi-level multiple logistic regression analyses of the Total satisfaction score

The multi-level multiple logistic analysis demonstrated that family members’ educational level, patients’ clinical treatment (number of psychologic treatments per day) and hospital-level data (doctor/bed ratio, nurse/bed ratio, and psychologist/bed ratio) were significantly associated with the total satisfaction score (see Table 4). For these analyses, we used the mean score as a cutoff score and categorized respondents scoring above and below that number into the satisfied and dissatisfied group, respectively. Other continuous variables were also transformed into appropriate categorical variables, based on quartiles and frequencies.

**Table 4:** Multi-level logistic regression examining related factors associated with total satisfaction score

Variables	Odds Ratio	95% CI (Lower)	95% CI (Upper)
Educational background (ref: Elementary school)			
Middle school**	1.86	1.33	2.60
High school**	2.08	1.46	2.97
College education**	2.22	1.56	3.14
Graduate education**	3.95	1.47	10.64
Treatment response (ref: Marked improvement)			
Improvement	0.72	0.13	4.03
Somewhat improvement	0.64	0.12	3.35
No change or worse	0.39	0.08	2.05
Self-payment (ref: ≤5000)			
5001–10,000	1.06	0.79	1.42
10,001–20,000	0.90	0.65	1.26
>20,000	0.81	0.56	1.16
Psychologic treatment times per day (ref: ≤0.40)			
0.41–1.0**	1.41	1.11	1.78
>1.0**	1.90	1.21	2.98
Doctor-bed-ratio (ref: ≤0.15)			
0.16–0.23	1.28	0.93	1.75
>0.23*	1.92	1.29	2.86
Nurse-bed-ratio (ref: ≤0.32)			
0.33–0.4*	1.53	1.06	2.21
>0.4**	4.46	3.03	6.55
Psychologist-bed-ratio (ref: ≤0.006)			
0.007–0.02	1.08	0.84	1.38
>0.02**	3.21	2.29	4.48

* *p*<0.05, ** *p*<0.001

Specifically, compared with participants with elementary school education or less, those with a middle school, high school, college, and graduate school educational background were more likely to be classified as satisfied (OR = 1.84, 2.08, 2.20, 3.92, respectively, all *p* < 0.001). Compared with family members of those patients who had received few psychological treatments (less than 0.4 times a day), family members of those who had received more psychological treatments were more likely to be classified as satisfied. Specifically, family members of patients receiving psychological treatments 0.41–1.0 times per day and > 1.0 times per day, were 1.4 times (OR = 1.40, *p* < 0.001) and 1.94 times more (OR = 1.94, *p* < 0.001) likely to be classified as satisfied. In hospitals where the doctor-bed-ratio was more than 0.23, the family satisfaction was 1.94 times greater than the satisfaction in the hospitals with a doctor-bed-ratio less than 0.15 (OR = 1.94, *p* < 0.05). In hospitals where the nurse-bed-ratio was 0.33–0.4 and more than 0.4, the family satisfaction was 1.51 and 4.32 times greater than the hospitals where the nurse-bed-ratio was less than 0.32 (OR = 1.51, *p* < 0.05; OR = 4.32, *p* < 0.001). When psychologist-bed-ratio was more than 0.02, family satisfaction was significantly higher than when the psychologist-bed-ratio was less than 0.006 (OR = 3.16, *p* < 0.001).

## Discussion

Family involvement and satisfaction in patient’s healthcare is particularly important in Chinese culture, as families are almost always involved in patient care, with few exceptions [32]. To the best of our knowledge, this is the first study to evaluate family member satisfaction with psychiatric inpatient services in a nationally representative sample. We found that the satisfaction level was overall high, although significant variations existed in different regions and among different hospitals. We found that higher educational background, more psychologic treatments offered per day, and optimal professional staffing (higher doctor/bed ratio, nurse/bed ratio, and psychologist/bed ratio) were all significantly related to higher family satisfaction.

In our study, the mean total satisfaction score was 23.46/25, or 93.84%. The result indicated that the families surveyed were very satisfied with the mental health inpatient services, which is consistent with some other studies [18, 19]. Gigantesco studied 105 relatives of psychiatric inpatients in Rome, Italy, and they reported a satisfaction score of 45.7/66, or 69.2% [17]. This difference may be due to the different demographic factors between the two samples. Since the Rome sample was from a predominantly urban area, the educational levels were quite modest; only 37% of the population had attained senior high school or greater levels of education. In our sample, the family members were from the capital city of each province, and 58.25% of them had a senior high school level or greater educational background. We found a higher education was significantly associated with higher satisfaction. As mentioned in the introduction, there have only been two reports on this topic published in China. Shi et al. (2004) reported the rate of satisfied or very satisfied family members was 77.1%, but due to differences in the instrument used, it is hard to compare it with our results. Another study focused on one aspect of psychiatric care. Specifically, they surveyed the satisfaction of nursing services [21].

We found that family total satisfaction score, and satisfaction scores for hospitalization cost and doctor-patient/family communication were higher than those reported by patients in this study. This result is consistent with findings both from China and other countries [17, 20, 33].

Our results showed that the education level of relatives was significantly associated with family satisfaction; higher education level was associated with greater family satisfaction. This is somewhat supported by another study [19], which showed that family caregivers with higher education levels had higher overall satisfaction scores (but it did not reach the statistical significance, possibly due to small sample size). This finding has also been reported in the non-psychiatric setting. For example, Thimmapur et al. studied caregiver satisfaction for intensive care unit services and found that caregivers with high school diplomas were more satisfied than illiterate caregivers [34]. Our findings differed from some other studies, which showed that lower education in families was significantly associated with higher satisfaction in relatives [17, 35, 36]. Many potential factors may contribute to this divergence, with one being mentioned in several studies—the service and decision-making model. Most believe that psychiatric services are more interactive and involve more shared decision making with family members; this may explain why educated family members would participate more readily and feel more satisfied [37–39].

We found that the provision of psychological treatment significantly contributed to family satisfaction, which is consistent with a previous report [23]. As patients and their family members are treated as customers [40, 41], and psychological treatment often focuses on listening and conflict resolution, it is not hard to understand why the number of psychological treatments for patients is significantly associated with higher satisfaction levels in both patients [23] and their families.

Not surprisingly, our findings also support a common-sense notion, that the adequacy of professional staffing is essential in service delivery and customer satisfaction. We found that higher ratios of psychiatric, psychologic, nursing staff, to bed number were all significantly associated with higher family satisfaction. While this finding is somewhat expected, it may increase awareness of this issue and motivate the government and hospital administrators to prioritize resources. Given that mental health service resources were overall insufficient and unbalanced across regions [42], prioritizing the hiring and retaining of professionals is essential.

It is worth noting that satisfaction is often affected by expectations. The high satisfaction in our study may have been due to family members’ low expectations. In the context of widespread discrimination against psychiatric patients and the poor public image of psychiatric hospitals, the patient expectation for mental health hospitals was assumed to be low [33, 43]. Measurements of expectations at baseline would help clarify this relationship.

A few limitations of this study need to be acknowledged. First, this is a cross-sectional study, which cannot uncover causal relationships. Second, the questionnaire we used in this study was locally developed, and the reliability and validity need to be more widely studied. Third, the samples were from provincial tertiary public psychiatric hospitals, and the generalizability may be limited. Forth, the data were collected on the day of discharge when both the patients and families were still at the hospital, which might lead to some measurement bias. Finally, a high degree of satisfaction (more than 90%) in our study implies a ceiling effect or/and high social desirability, suggesting a more sensitive assessment tool or an additional tool to measure social desirability may be needed in future studies.

## Conclusions

In conclusion, we developed a brief and reliable instrument to measure the satisfaction level of family members of psychiatric inpatients. Our results show that the family satisfaction with inpatient psychiatric services overall was high in China, although significant variations existed across different regions and among different hospitals. Overall, family members seemed to be more satisfied than patients with the inpatient service. Several factors related to individual, treatment, and hospital resources were found to be significantly associated with the satisfaction of family members. Notably, the education level of the participants, psychological treatment patients received, and adequacy of mental health professional staffing were all significantly associated with higher satisfaction. These results highlight for policymakers and hospital administrators a number of opportunity areas to improve family members’ satisfaction.

## Data Availability

Because of ownership of the data and the local policy, we do not wish to share data.

## Abbreviation

CGI: Clinical Global Impression
ECT: Electroconvulsive therapy
GAF: Global Assessment of Functioning
HIS: Hospital Information System
ICD-10: the International Classification of Diseases and Related Health Problems 10th revision
IQR: Inter-quartile range
IRB: Institutional Review Board
LOS: Length of stay (days)
OR: Odds ratio
WHO: World Health Organization
